# Dynamic PET/CT with the Hepatobiliary Tracer [68Ga]Ga-Tmos-DAZA for Characterization of a Hepatic Tumor

**DOI:** 10.3390/diagnostics11040660

**Published:** 2021-04-06

**Authors:** Martin Freesmeyer, Julia Greiser, Thomas Winkens, Falk Gühne, Christian Kühnel, Falk Rauchfuß, Hans-Michael Tautenhahn, Robert Drescher

**Affiliations:** 1 Clinic of Nuclear Medicine, Jena University Hospital, Am Klinikum 1, 07747 Jena, Germany; julia.greiser@med.uni-jena.de (J.G.); thomas.winkens@med.uni-jena.de (T.W.); falk.guehne@med.uni-jena.de (F.G.); christian.kuehnel@med.uni-jena.de (C.K.); robert.drescher@med.uni-jena.de (R.D.); 2 Clinic of General, Visceral and Vascular Surgery, Jena University Hospital, Am Klinikum 1, 07747 Jena, Germany; falk.rauchfuss@med.uni-jena.de (F.R.); hans-michael.tautenhahn@med.uni-jena.de (H.-M.T.)

**Keywords:** DAZA, dynamic PET, liver, HCC, hepatobiliary function, gadoxetic acid

## Abstract

Established imaging modalities for the characterization of liver tumors are computed tomography (CT), magnetical resonance (MR) imaging, sonography, and hepatobiliary scintigraphy. In some cases, their results may be inconclusive or certain examinations not possible due to contraindications. Positron emission tomography (PET)/CT has the capability of dynamic imaging with high temporal resolution. With radiolabeled tri-alkoxysalicyl-1,4-diazepan-6-amine (TAoS-DAZA) tracers, imaging of liver perfusion and hepatobiliary function is possible in a single examination. In the presented case, the PET/CT was performed in a patient with suspected hepatocellular carcinoma and atypical CT findings. PET imaging characteristics were consistent with a hepatocellular carcinoma (HCC). PET with DAZA ligands may be a supplemental method for liver tumor characterization in difficult cases.

Characterization of tumors is a prerequisite of successful treatment, and is mostly done by biopsy and histopathological examination. A major exception is HCC, which is the most common liver cancer. In many cases, a diagnosis is established based on imaging alone, because there is a risk of extrahepatic tumor spread caused by biopsy. Several imaging criteria guidelines have been established, including non-rim arterial phase contrast media hyperenhancement and washout in the portalvenous phase. In some guidelines, the latter criterion is extended to the hepatobiliary phase [1]. However, in some clinical cases MR imaging, which is the best modality for liver tumor evaluation, is not possible due to contraindications, and other options are required (Figure 1, Figure 2, Figure 3 and Figure 4).

The case shows that PET imaging with DAZA ligands may be evolve to be a supplemental method for liver tumor characterization in difficult cases, combining information about tissue perfusion with those of hepatobiliary function and excretion.

## Figures and Tables

**Figure 1:**
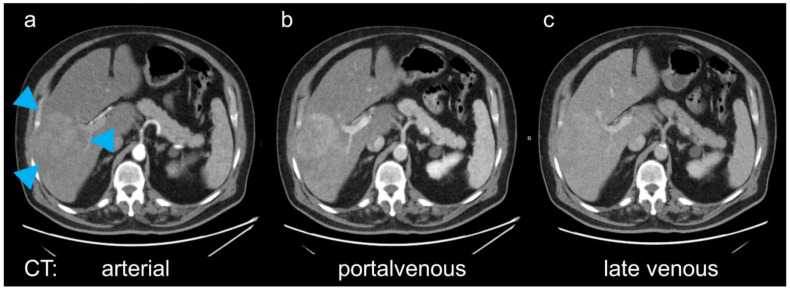
In the presented patient, on computed tomography (CT) the mass in the right liver lobe (a, arrowheads) showed arterial (**a**) and portalvenous (**b**) enhancement, with a mixed appearance on the late venous phase with slightly hyper-, but also hypodense areas (**c**). The diagnostic dilemma was that a biopsy should be avoided, and the patient could not undergo magnetical resonance (MR) imaging because of a pacemaker. Fast dynamic hepatobiliary scintigraphy is limited to planar acquisition, and not feasible for the evaluation of focal lesions [2].

**Figure 2:**
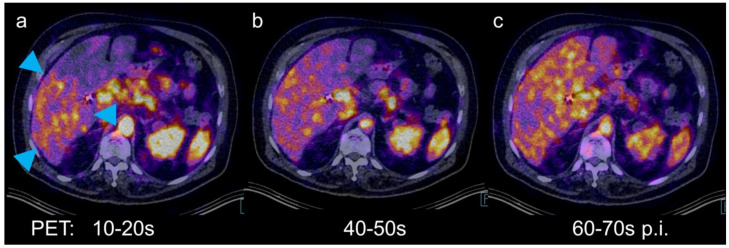
Positron emission tomography (PET)/CT has dynamic imaging capabilities for the depiction of liver tumors [3]. Therefore, an examination with the hepatobiliary tracer [68Ga]Ga-tri-methoxysalicyl-(TMoS)-DAZA was performed. Similar to mebrofenin derivatives and liver-specific MR contrast agents (e.g., gadoxetic acid), this 1,4-diazepan-6-amine (DAZA) ligand is extracted from the blood by hepatocytes and secreted into the bile [4,5,6]. In this case, 155 MBq of the tracer were injected intravenously. A dynamic PET of the liver over 3 min was acquired and reconstructed in 10-s timeframes. Early arterial hyperperfusion was evident in the region of the tumor (**a**, arrowheads). Tumor uptake was equal to normal liver 50 s after injection (**b**). From 60 s after injection, uptake in the tumor was lower than in the surrounding liver tissue (**c**).

**Figure 3:**
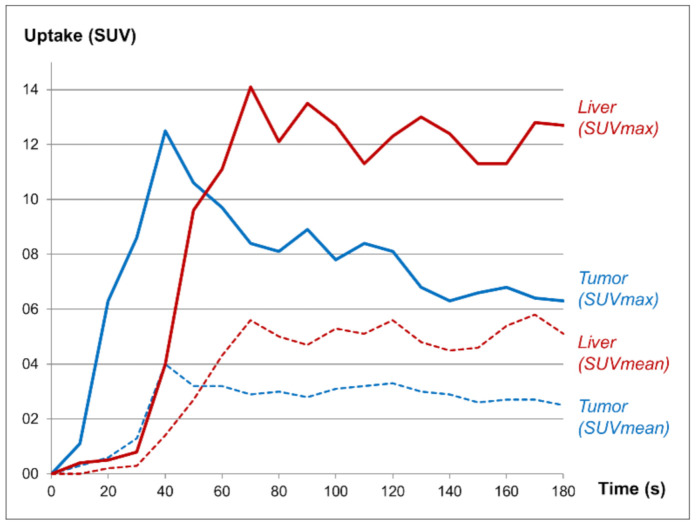
Measurements of standardized uptake values (SUV) in the tumor and in a liver segment not involved by tumor showed fast tumor perfusion followed by a “washout” of tracer (blue curves), while the liver uptake remained relatively steady (red curves).

**Figure 4:**
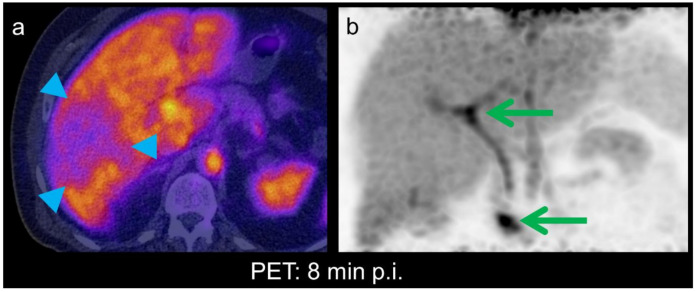
Liver function in a hepatocellular carcinoma (HCC) depends on the degree of dedifferentiation, substrate uptake, processing and bile excretion still taking place in well and moderately differentiated HCCs [7]. On PET/CT acquired in the hepatobiliary phase, eight minutes after injection, tumor uptake was approx. In this case, 50% of normal liver tissue, consistent with reduced but not absent cellular function (**a**, arrowheads). Tracer excretion into bile ducts and duodenum can be seen on the corresponding maximum intensity projection (MIP) image (**b**, green arrows). The tumor itself is barely visible due to the overlying liver tissue. In summary, the features were suggestive of an HCC. The patient underwent surgery. Histopathological examination showed a moderately differentiated HCC.
